# Cold Plasma Deposition of Tobramycin as an Approach to Localized Antibiotic Delivery to Combat Biofilm Formation

**DOI:** 10.3390/pathogens13040326

**Published:** 2024-04-16

**Authors:** Beatrice Olayiwola, Fiona O’Neill, Chloe Frewen, Darren F. Kavanagh, Rosemary O’Hara, Liam O’Neill

**Affiliations:** 1 Department of Science and Health, South East Technological University, Kilkenny Road, R93 V960 Carlow, Irelanddarren.kavanagh@setu.ie (D.F.K.); rosemary.ohara@setu.ie (R.O.); 2 TheraDep Inc., 2200 Zanker Road, San Jose, CA 95131, USA

**Keywords:** cold atmospheric plasma, tobramycin, coating, antibiotic efficacy, biofilm formation, titanium

## Abstract

Hospital-acquired infections (HAIs) remain a significant factor in hospitals, with implant surfaces often becoming contaminated by highly resistant strains of bacteria. Recent studies have shown that electrical plasma discharges can reduce bacterial load on surfaces, and this approach may help augment traditional antibiotic treatments. To investigate this, a cold atmospheric plasma was used to deposit tobramycin sulphate onto various surfaces, and the bacterial growth rate of *K. pneumoniae* in its planktonic and biofilm form was observed to probe the interactions between the plasma discharge and the antibiotic and to determine if there were any synergistic effects on the growth rate. The plasma-deposited tobramycin was still active after passing through the plasma field and being deposited onto titanium or polystyrene. This led to the significant inhibition of *K. pneumoniae*, with predictable antibiotic dose dependence. Separate studies have shown that the plasma treatment of the biofilm had a weak antimicrobial effect and reduced the amount of biofilm by around 50%. Combining a plasma pre-treatment on exposed biofilm followed by deposited tobramycin application proved to be somewhat effective in further reducing biofilm growth. The plasma discharge pre-treatment produced a further reduction in the biofilm load beyond that expected from just the antibiotic alone. However, the effect was not additive, and the results suggest that a complex interaction between plasma and antibiotic may be at play, with increasing plasma power producing a non-linear effect. This study may contribute to the treatment of infected surgical sites, with the coating of biomaterial surfaces with antibiotics reducing overall antibiotic use through the targeted delivery of therapeutics.

## 1. Introduction

Implant-associated infections (IAIs) are a frequent problem in the healthcare industry due to the fact that implants can become colonized with microbes after surgery, which can lead to significant complications. The bacteria involved in IAIs are typically the ESKAPE pathogens, which are *Enterococcus faecium*, *Staphylococcus aureus*, *Klebsiella pneumoniae*, *Acinetobacter baumannii*, *Pseudomonas aeruginosa* and *Enterobacter species*. These pathogens are increasingly difficult to treat due to their resistance to antibiotics, which makes the biofilm layer that they create on the implant surfaces harder to eradicate [1,2]. Up to 60% of general bacterial infections and 80% of chronic infections are due to the proliferation of bacterial colonies within the protective biofilm structure [3]. Biofilms can attach to medical devices such as orthopaedic implants, central venous catheters, and urinary catheters [4].

These biofilms consist of unattached and aggregated cell biomass that can develop on the surface of the implants and evade the host’s immune system [5]. This biomass is then encased within a self-produced matrix that comprises an extracellular polymeric substance (EPS). This EPS layer provides protection for the biofilm and acts as a shield that maintains the structural integrity of the biofilm intact [6]. These biofilm structures can aid the survival of the microorganism as they are resistant to external stressors and can protect against antibiotic stressors. This can lead to regular antibiotic treatment being ineffective against bacterial infection. As a consequence, up to 80% of biofilm-related infections do not respond to antibiotic treatment [3]. For example, *Klebsiella pneumoniae* (*K. pneumoniae*) is a Gram-negative encapsulated non-motile bacterium that causes infections in hospital settings, such as pneumonia, bacteraemia, urinary tract, and wound infections [7]. *K. pneumoniae* is an opportunistic multi-drug-resistant (MDR) pathogen that possesses many mechanisms of resistance to protect itself from the host immune system [8].

Some of these resistance mechanisms include efflux pumps, which allow the pathogen to regulate the internal environment by removing the toxic substances [9]. The efflux pump is said to contribute to resistance towards β-lactam drugs as well as quinolones and macrolides [10]. Fortunately, aminoglycoside tobramycin can be used to treat *K. pneumoniae* infections and acts by disrupting the replication of the bacteria by binding to the RNA, leading to mistranslation and death due to a non-functional bacterial cell.

An alternative method of treating these resistant strains of bacteria is through the use of cold atmospheric plasma (CAP). CAP is generated by applying radio frequency energy to gases, thereby producing an ionised glowing electrical discharge. This discharge is filled with a variety of active species, including ions, electrons, free radicals, ultraviolet radiation, and a wide variety of reactive oxygen and nitrogen species that can degrade and kill bacterial cells using a variety of mechanisms. This can include the oxidation of membrane proteins, direct perforation of the cell membrane, and the oxidation of the key proteins within the cell. The plasma may also disrupt nucleotide strands through a combination of UV-induced damage and oxidative chemical effects [11]. As such, there are no known bacterial resistance mechanisms to CAP treatment and it has a wide spectrum of activities [12,13]. CAP has also been observed in a study carried out by Scholtz et al., 2021, to have antimicrobial properties against biofilm structures [14], though the biofilm does provide significant protection to the bacteria contained within [11].

Plasma can also be used to deposit antibiotics onto implant surfaces instead of the wet chemistry methods that are used in industry today [4,15]. The plasma and antibiotic combination can allow for the coating of an antibiotic on the surface without the use of polymers or binders, which can thus improve the biocompatibility of the antibiotic. The presence of an antibiotic layer on a surface can also prevent aggregated biomass formation and reduce the likelihood of infection on the implant surface. In this study, the interaction of *K. pneumoniae* in its planktonic and biofilm form was observed on the tobramycin-coated surface of titanium coupons and also polystyrene plates. The aim of this study was to observe whether a high bacterial load of *K. pneumoniae* in its exponential growth phase could be decreased within six hours after incubation on a tobramycin-coated surface. Additionally, the study set out to determine whether exposed *K. pneumoniae* biofilms could be reduced using CAP and tobramycin both separately and combined.

This research has the potential to decrease the likelihood of implant-associated infections (IAIs) as well as the side effects associated with the infections. The use of CAP as a pre-treatment followed by antibiotics can potentially prevent and reduce the growth of biofilm structures at surgical sites and on implant surface [16,17].

## 2. Materials and Methods

### 2.1. Bacterial Strain Preparation

The *Klebsiella pneumoniae* WDCM 0097 vitroids were obtained from Sigma Aldrich^®^, Darmstadt Germany, and were rehydrated and cultured as per the manufacturer’s specifications. The stock cultures used had a starting concentration of up to 1 × 10^10^ CFU/mL, and bacterial growth was monitored using the standard plate method using the Accuris microplate SmartReader 96™, Accuris instruments, Edison, NJ, USA.

The stock solutions were sub-cultured in Tryptone Soya Broth (TSB) and incubated for 24 h at 37 °C. CFU/mL was calculated by streaking dilutions of the inoculum onto Tryptone Soya Agar (TSA) and incubating it for 24 h followed by counting the colonies formed.

### 2.2. K. pneumoniae Biofilm Growth on Microtiter Polystyrene Plates

An inoculum was prepared using a 24 h broth that contained 10^8^ CFU/mL. This broth was diluted to obtain a 10^6^ CFU/mL culture. The inoculum was then pipetted into the wells of microtiter 96-well Sarstedt plates, and the volume pipetted was 200 µL. The culture was then incubated at 37 °C for 4 days to obtain the formed biofilm structures.

### 2.3. Preparation of Stock and Working Solution of Tobramycin

Tobramycin Sulphate (Avantor) was stored as per the manufacturer’s specifications. For minimum inhibitory concentration (MIC) testing, a 10 mg/mL solution was prepared in sterile deionized water, and the working solutions of 1 mg/mL and 0.1 mg/mL were diluted in TSB. For plasma deposition onto titanium coupons, three different working solutions were prepared in sterile deionized water, which were 10, 15, and 20 mg/mL. For plasma deposition onto polystyrene 96-well plates, antibiotic solutions (at concentrations of 10 mg/mL and 15 mg/mL) were prepared using sterile deionized water.

### 2.4. Measurement of Absorbance

To monitor the growth of the planktonic and biofilm formation, optical density was read using a Accuris SmartReader 96 at 630 nm. Planktonic cell growth was monitored during a 6 h time point, and the biofilm formation post plasma deposition treatment was observed after 6 days of incubation.

### 2.5. Crystal Violet Assay on the Formed Biofilms

A crystal violet (CV) assay was carried out at room temperature, as described by Los et al., with minor modifications [4]. After four days, the planktonic cell supernatant was removed from the wells to expose the formed biofilms, and 100 µL of 99% methanol was added into each of the wells.

The 99% methanol remained for 15 min and was then removed; the plates were air-dried, and 100 µL of the 0.2% crystal violet solution (CV) was added. The 0.2% solution was made by diluting 200 µL of pure crystal violet solution (100%) into 100 mL of methanol. The CV solution was removed after 20 min, and the wells were rinsed with 150 µL of phosphate-buffered saline (PBS). After the PBS rinse, the remaining liquid was discarded and, finally, 0.33% acetic acid was added into the wells to remove any unreleased CV. After 15 min, the acetic acid solution was pipetted into a new plate.

The plates were then read at 630 nm, and the blank (acetic acid) was subtracted from all of the sample absorbances to obtain the absolute value.

### 2.6. Antibiotic Susceptibility of K. pneumoniae (MIC)

MIC testing was conducted by using a range of different tobramycin concentrations on the same CFU/mL of the *K. pneumoniae* stock. Determination was carried out following the recommended testing procedure of the Clinical and Laboratory Standards Institute protocols (CLSI, 2012) and according to the protocol of Los et al., 2019 [18,19].

Then, 100 µL tobramycin solution was diluted in TSB, creating a 1 mg/mL solution, which was then pipetted in triplicate into wells A1 to A3, in the 96-well plate (Sarstedt). In all of the rows listed below (B1 to H1, B2 to H2, and B3 to H3), 50 µL of TSB (no antibiotic) was added. A concentration gradient was created by pipetting 50 µL of the tobramycin stock from the first 3 wells (A1 to A3) into the following row below (B1 to B3); this was continued down in a row-to-row manner all the way to H1 to H3, creating a broth micro-dilution. Finally, 50 µL of *K. pneumoniae* (10^6^ CFU/mL) was inoculated into each well (rows A1 to H1, A2 to H2 and A3 to H3), and a final concentration of 3 × 10^5^ CFU/mL was obtained.

The negative control in this experiment was TSB only, and the positive control was *K. pneumoniae* growth (10^6^ CFU/mL). An additional control was the TSB and tobramycin stock to ensure that the antibiotic did not have a negative effect on the TSB absorbance reading. All of the inoculated plates and the TSB control were incubated for 24 h at 37 °C. Bacterial growth was then observed spectrophotometrically after 24 h using microplate reader Accuris SmartReader 96 at 630 nm. MIC was defined as the lowest amount of the antibiotic solution needed to inhibit the growth of the *K. pneumoniae* culture [20].

### 2.7. Plasma Deposition of Tobramycin onto Polystyrene 96-Well Plates and on Titanium Coupons

**Figure 1:**
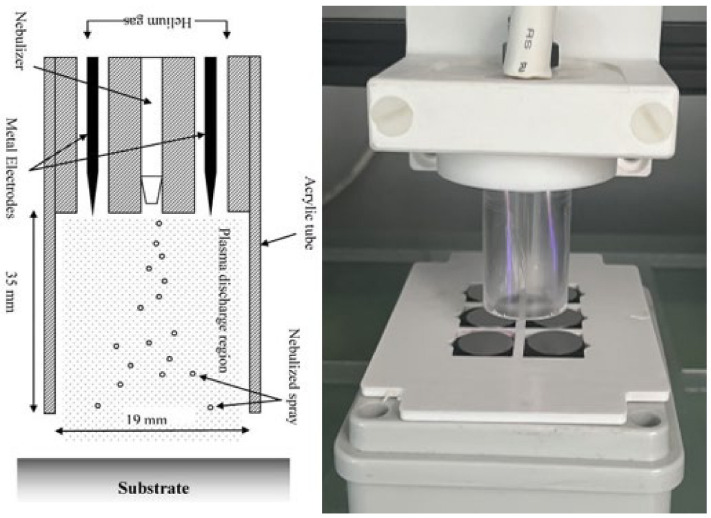
The figure shows the system diagram (**left**) and image (**right**) of the BioDep plasma deposition system [4,21]. Unit powered by high-voltage power supply that provides RF voltage at 20 kHz to the two electrodes creating electric field. The nebulized antibiotic particles deposited onto titanium coupons using pneumatic nebulizer (left image provided by Theradep Ltd.).

The deposition was carried out using a BioDep plasma unit (TheraDep Ltd., Clonmel, Tipperary, Ireland) purpose-built deposition system that generates it power using the Redline G2000 High-Voltage power supply (Figure 1). A Teflon deposition unit is connected to the power supply, which is comprised of two electrodes and a nebulizer (Burgener, T2100) that is connected to the syringe pump. The syringe pump holds the liquid of interest, which is set at a particular flow rate that creates a constant deposition flow of the nebulized antibiotic liquid.

To generate plasma discharge, helium gas and high-voltage power were fed into the Teflon head, where the gas flows around the electrodes and creates plasma. The nebulized antibiotic spray and the plasma discharge were all contained within an acrylic tube (18 mm diameter × 35 mm in length). The substrates to be coated were at the outlet of the tube, and the Teflon unit was controlled using a numerically controlled table and moved in a raster pattern over the substrate.

#### 2.7.1. Coating for the Planktonic Growth Observation over 6 h

To observe planktonic cell growth, the microtiter plates were plasma-cleaned and activated prior to deposition, with the exception of the control and wet deposition control, which received no plasma treatment. This activation was carried out using a Diener Zepto vacuum plasma chamber with helium as a feed gas for 1 min. The flow rate of the feed gas entering the chamber was 0.5 L/h, and the chamber was set to 100% power.

The deposition of tobramycin on the 96-well plates was carried out using a liquid concentration of 15 mg/mL of tobramycin. A flow rate of 60 µL/min was used to deposit the antibiotic in three layers. This was carried out for non-plasma deposition (using no plasma) and plasma deposition using 90 V of plasma. The control consisted of an inactivated plate with no plasma or antibiotic deposition whatsoever. Additionally, non-plasma antibiotic deposition was carried out using an inactivated plate; this was considered the ‘non-activated wet deposit’.

#### 2.7.2. Deposition onto *Klebsiella pneumoniae* Biofilms

For biofilm formation via the plasma deposition of tobramycin, the microtiter plates were not activated using plasma. The wet deposit parameter consisted of three sections. Section 1 was treated using a flow rate of 60 µL/min of antibiotic. The subsequent section was treated with an 80 µL/min flow rate of antibiotic, and the last section was treated with a 100 µL/min flow rate of antibiotic solution. All of these sections were carried out on the developed *K. pneumoniae* biofilm after 4 days.

For the plasma and antibiotic treatments, an initial plasma treatment was used on the biofilms, followed by the deposition of the antibiotic solution at 60 µL/min. The three parameters included the use of 90, 100, and 110 V of plasma followed by the deposition of the antibiotic solution.

Lastly, a plasma-only treatment was carried out by applying 90, 110 and 120 V separately to 3 sections of the plate with the formed biofilms. The treatment for each section lasted for 2 min and 30 s.

#### 2.7.3. Coating of Titanium Surface with Tobramycin

The titanium coupons were activated prior to deposition. This activation was carried out using a Diener Zepto vacuum chamber with helium used as the feed gas for 1 min. The flow rate of the feed gas entering the chamber was 0.5 L/h, and the chamber was set to 100% power.

For the titanium coupons, different tobramycin concentrations were deposited onto the coupons. The deposited tobramycin was deposited with and without plasma discharge. Non-plasma deposition was referred to as ‘wet deposition’, and these wet samples were allowed to air dry overnight before further testing. This was carried out for the 10, 15, and 20 mg/mL tobramycin solutions.

For the second aspect of the coupon coating, 10 mg/mL solution was used to coat the coupons in layers. Three parameters were used by layering the antibiotic with 1, 2, and 3 layers of the tobramycin solution over the coupons. After the plasma and non-plasma depositions, the microtiter plates used for the planktonic study and coupons were vacuum-sealed and stored at room temperature.

### 2.8. Planktonic K. pneumoniae Growth on Tobramycin Coated 96-Well Plates

Tobramycin-coated microtiter plates were used to evaluate the planktonic cell growth using 200 µL of *K. pneumoniae* in TSB (1 × 10^6^ CFU/mL) per well. This concentration of *K. pneumoniae* was used in order to mimic the exponential growth phase of the bacteria and allow for the study of treating live cells rather than a few live or dead cells [22]. These volumes of bacteria were pipetted into three rows of the wet deposits and plasma deposits as the negative control plates. The positive controls were the non-activated wet deposits and inactivated plate (no antibiotic or plasma treatment), which contained 200 µL of the bacterial culture.

These plates were then incubated at 37 °C for a defined time period of 0, 2, 4, and 6 h. At each time point, the plates were removed from the incubator, and the absorbance of planktonic cell growth was measured at 630 nm. The averages of the absorbance were acquired, and the CFU/mL was calculated at each time point using the standard plate count method.

### 2.9. Treatment of K. pneumoniae Biofilms Using CAP and Tobramycin in 96-Well Plates

The bacterial inoculum was pipetted into four uncoated Sarstedt plates, and the biofilms were grown at 37 °C for 4 days. After this incubation period, the inoculum was pipetted out of all the plates, and the wells were rinsed once with 100 µL of phosphate-buffered saline (PBS) and left to dry for 20 min.

Once dry, the plates were divided into the following: blank, plasma-treated, wet deposit, and plasma treatment followed by antibiotic deposition. The blank plate, which was not treated with any antibiotic or plasma, acted as a control. The sections of each parameter were treated and coated as per described in Section 2.7.

After all the plate treatments, 200 µL of fresh TSB was added back into all of the wells, including the blank plate and the plates, and were incubated for a further 2 days at 37 °C. After the 2 day period, total biofilm formation was quantified using the CV assay, and the absorbance was measured at 630 nm on the Accuris SmartReader 96™.

### 2.10. Efficacy of Tobramycin-Coated Titanium Coupons

All of the titanium samples were placed onto Mueller–Hinton agar plates that were inoculated with 1 × 10^6^ CFU/mL of *K. pneumoniae in* triplicate.

Once these coupons were placed, they were left to fixate to the inoculated plates before being transferred into the incubator set to 37 °C for 24 h. The zones of inhibition (ZOI) created by the antibiotic-coated coupons and controls were measured the following day.

### 2.11. Contact Angle

The TVA100 contact angle system (Kruss, Nurnburg, Germany) was used to observe the changes to the substrate surface before and after the coating, activation, and coating of the substrate. Contact angle (CA) analysis was carried out using dyed deionized water pipetted onto the substrate surface in small quantities (0.5 µL).

Once the dyed water was on the surface, the CA was measured using top view analyser, and the images were recorded at 50 frames per second (fps). Each sample was measured in triplicate after 1 min of contact with the surface. These data were recorded and analysed using the Kruss Advance software (version 1.12).

### 2.12. FTIR

FTIR (Fourier transform infrared) spectroscopy was carried out to directly measure the coated sample by depositing tobramycin sulphate directly onto NaCl discs. The tobramycin was deposited at 60 µL/min using a 20 mg/mL solution. The dissolved antibiotic was deposited onto PerkinElmer NaCl discs that was enclosed in a card.

The wet-deposited sample (non-plasma) sample was produced using plasma as a form of static deposition for 1 min.

### 2.13. Atomic Force Microscopy

Atomic force microscopy (AFM) was carried out using NanoSurf EasyScan (Liestal, Switzerland), with a z height of 4.9 µm and a tip voltage of 0 V. Overscan was enabled at 10%, and the scan range was 50 µm. The topography of each sample and the analysis data of the surface were taken in triplicate. These data were then analysed using Nanosurf Easyscan 2 software (version 3100).

A blank titanium coupon with no coating and plasma treatment was also scanned in triplicate as a reference. The samples were coated as per the procedure described in Section 2.7, ‘Plasma deposition of tobramycin on polystyrene 96-well plates and on titanium coupons’. The plasma and non-plasma-deposited coupons were analysed at each concentration: 10, 15, and 20 mg/mL.

## 3. Results

### 3.1. Susceptibility of the K. pneumoniae against the Tobramycin

**Figure 2:**
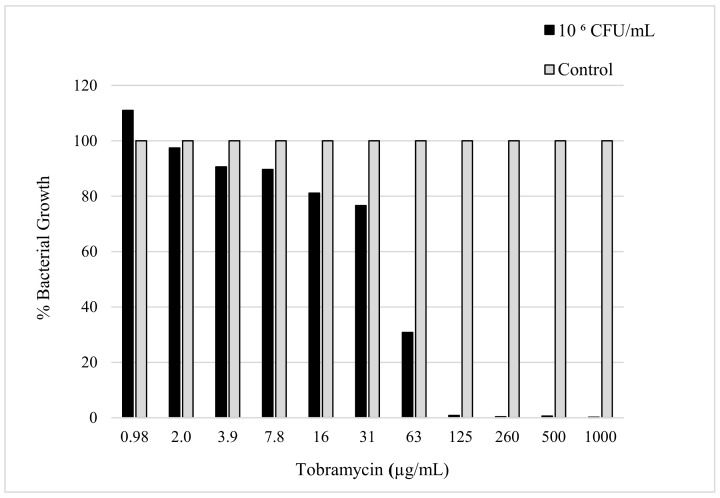
The figure shows susceptibility testing of tobramycin sulphate against *K. pneumoniae* culture (10^6^ CFU/mL) measured in bacterial growth (%) acquired by obtaining the absorbance (630 nm) after 24 h incubation.

The sensitivity of *K. pneumoniae* to tobramycin was confirmed by determining the minimum amount of antibiotic needed to reduce the bacterial load. The evaluation of the MIC was determined using the micro broth dilution method, which was carried out with consideration of the CLSI and European Committee on Antimicrobial Susceptibility Testing (EUCAST) guidelines [19]. The MIC value can be seen in Figure 2, which shows the minimum amount of tobramycin required to inhibit the bacterial growth of a 50 µL sample with a 10^6^ CFU/mL concentration of *K. pneumoniae.* The MIC was observed to be 125 µg/mL after a 24 h incubation period with the antibiotic in the bacterial culture.

The MIC was used to determine the minimum amount of antibiotic needed to prevent bacterial growth. This value was used to determine the concentration of tobramycin to deposit in order to observe the changes in growth depending on the parameter applied to the *K. pneumoniae* culture.

### 3.2. The Growth Rate of Planktonic K. pneumoniae Bacterial Colonies on Tobramycin Coated 96-Well Plates

**Figure 3:**
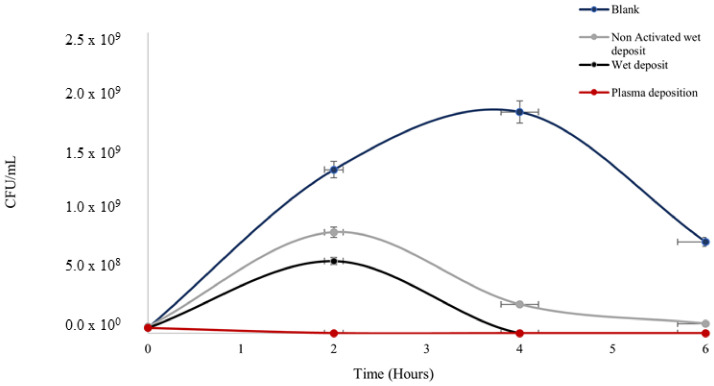
The figure shows *Klebsiella pneumoniae* growth (CFU/mL) over the span of 6 h on coated 96-well polystyrene plates; blank wet deposition (non-plasma, activated plate (positive control)), non-activated wet deposit (NAWD), and plasma deposition (activated plate (positive control). Tobramycin deposition at 15 mg/mL, 3 layers and plasma deposition at 90 V. See Table 1 for *t*-test analysis.

The plasma-deposited tobramycin on polystyrene plates proved to be effective in reducing the population of the planktonic cells of *K. pneumoniae* using the standard plate counting method. The *K. pneumoniae* colonies started growth in the exponential phase, which was estimated using a standard bacterial growth phase graph [22]. The starting bacterial concentrations at 0 h across all the parameters (Figure 3) were 1 × 10^7^ CFU/mL, which correlated to the exponential growth phase on the graph curve.

Antibiotic efficacy was retained on the surface after deposition with the plasma and wet deposition, showing the lowest rate of colonies (Figure 3) in comparison to negative control 1 (blank: no tobramycin coating), which had a prominent level of colony growth at 1 × 10^9^ CFU/mL.

The blank control peaked at the 4 h time point before reducing by the 6 h time point. The starting concentration of the culture was 1 × 10^6^ CFU/mL at 0 h, which shows that at 4 h, the culture entered the stationary phase before the death phase at the time point of 6 h.

Bacterial growth on the non-activated wet deposit plate (negative control 2) was slightly higher, with an increase of up to ten times in terms of CFU/mL when compared to the wet and plasma-deposited plates, as there was evidence of activation on these positive controls. This displayed that the plasma activation on the positive control plates prior to deposition retained the antibiotic on the surface more efficiently than the inactivated, non-activated wet deposit plates. Ultimately, the blank control appeared to remain in the stationary phase of growth longer than the treated samples, which displayed a lower level of CFUs.

**Figure 4:**
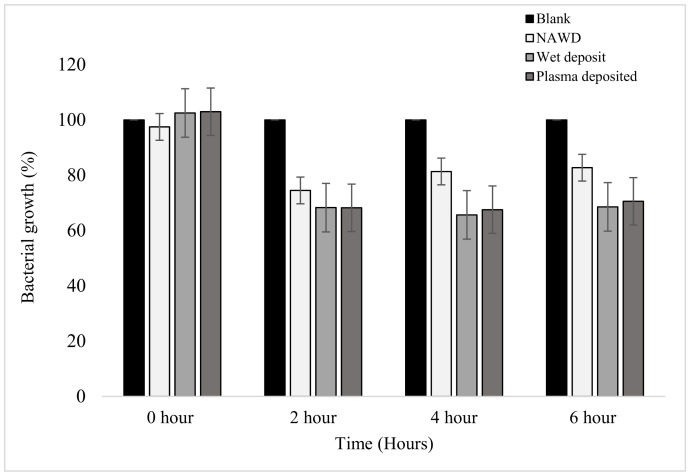
The figure shows the bacterial growth of *K. pneumoniae* over a period of 6 h based on absorbance at 630 nm on coated 96-well plates. The samples were as follows: blank (no antibiotic), non-activated wet deposited (antibiotic deposited without plasma on a non-activated plate (positive control)), wet deposited (antibiotic sprayed without plasma on an activated plate), and finally, plasma-deposited (combination of plasma and antibiotic (positive control)). Tobramycin deposition at 15 mg/mL, 3 layers (*n* = 16).

Similarly, when the absorbance of the planktonic cells was measured at each time point, it could be seen that the results mirrored that of the standard plate counting method displayed above. In Figure 4, the positive controls (wet deposit and plasma deposit on an activated plate) and negative control 2 (NAWD) were effective in reducing the bacterial growth of the planktonic bacterial colonies when compared to the blank (negative control 1).

A steady decrease in bacterial growth was observed at the 2 h time point, with the positive controls reducing by up to 32%. The non-activated wet deposit was reduced by only 26% at the 2 h time point, which suggests that the inactivated surface retained less antibiotic when compared to the positive controls. The plasma-deposited samples have a similar effect to the wet deposit sample at all time points. This slight difference shows that tobramycin remained active despite being exposed to the plasma during deposition.

From the 2 h to the 4 h time point, the bacterial growth of the wet deposited sample decreased stably by 2.6%, and the plasma-deposited sample decreased by 0.7%, showing no meaningful change. These positive controls remained stationary, with little to no further bacterial growth. However, in the non-activated wet deposited sample, it could be seen that bacterial growth increased by 6.9% from 2 to 4 h.

Furthermore, *t*-test analysis was carried out to demonstrate the difference between time points at which the planktonic *K. pneumoniae* was observed in terms of growth. There were no initial differences between the blank sample and the parameters listed (Table 1). As the number of hours increased, there was a significant difference between blank sample growth in comparison to the treated samples at 2, 4, and 6 h, and the difference was statistically significant (*p*-values < 0.05). Overall, the greatest significance can be observed at the 2 h mark, as it could be seen that the *p*-values were extremely low, displaying the effect of the treatments on the planktonic *K. pneumoniae* sample in comparison to the untreated sample.

### 3.3. The Efficacy of the Tobramycin-Coated Titanium Coupons on K. pneumoniae

**Figure 5:**
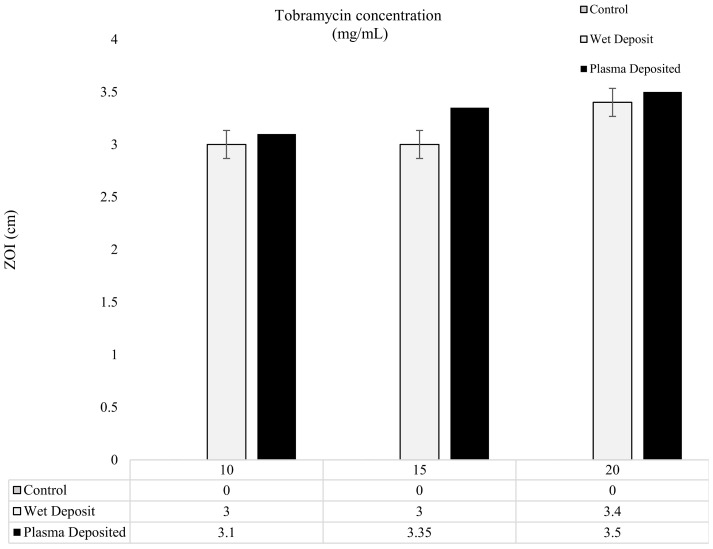
The figure shows tobramycin-coated coupons (plasma and wet deposit) at varying concentrations (10, 15, and 20 mg/mL) plated onto 10^6^ CFU/mL of *K. pneumoniae* and the zones of inhibition (ZOI) created measured in cm (*n* = 3).

**Figure 6:**
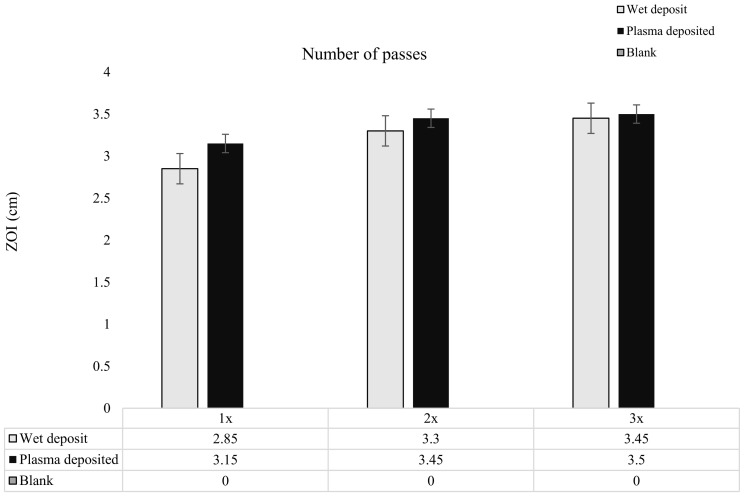
The figure shows tobramycin-coated coupons at varying concentrations through increasing the number of passes of tobramycin (10 mg/mL) over the coupon surface. Zones of inhibition (ZOI) created through plating the coupons on the lawn if 10^6^ CFU/mL of *K. pneumoniae* on MHA (*n* = 3).

The results of the modified Kirby–Bauer method are depicted in Figure 5 and Figure 6, showing the level of antibiotic present in the form of zones of inhibition (ZOI). In Figure 5, the varying concentrations of tobramycin were deposited on the titanium coupons to produce a single layer of coating, and it could be observed that the zones created gave comparable results for the 10, 15, and 20 mg/mL coupons. The plasma-deposited coupons produced a slightly larger zone, but the effect was relatively similar to that of the wet-deposited samples. The wet-deposited ZOI remained the same at 10 and 15 mg/mL and produced an increase of 0.4 cm at 20 mg/mL.

On the other hand, the plasma deposit sample displayed an increase of up to 0.25 cm from the 10 mg/mL to 15 mg/mL samples and a 0.15 cm increase from the 15 mg/mL to the 20 mg/mL samples. The slight differences between the plasma-deposited and the wet-deposited samples showed that the plasma did not have a negative effect on antibiotic functionality. Despite the 5 mg/mL increases in the antibiotic concentration, the ZOI remained relatively similar, with no major changes being observed.

As the tobramycin deposition was layered, it was observed that there was a slight increase in the ZOI from pass 1 to pass 3 for both the wet-deposited and plasma-deposited sample. The wet-deposited sample showed an increase of up to 0.24 cm from pass 1 to pass 2 and 0.15 cm from the 2 passes to the 3 pass layers. These increases were like that of plasma deposition, which displayed an increase of 0.3 cm from pass 1 to pass 2 and 0.05 cm from pass 2 to pass 3.

The increase in antibiotic concentration through layering, shown in Figure 6, did not significantly change the ZOI created on the *K. pneumoniae* lawn. The coated coupons retained antibiotic activity, with the plasma and wet-deposited samples creating a long-lasting bactericidal effect.

### 3.4. The Efficacy of Plasma Treatment in Conjunction with Tobramycin to Treat Fully Formed K. pneumoniae Biofilm

**Figure 7:**
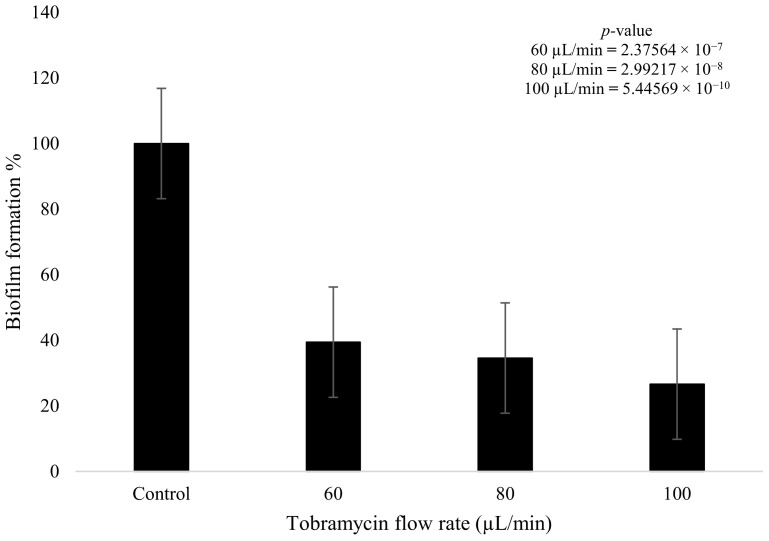
*K. pneumoniae* biofilm growth (%) after tobramycin (15 mg/mL) wet deposition at 60 µL/min, 80 µL/min, and 100 µL/min (*n* = 32). *p*-value results of the treatments when compared to the control sample that consisted of no treatment included in top right corner.

Tobramycin and plasma were used to treat the fully formed *K. pneumoniae* biofilms after 4 days of growth. The tobramycin wet deposit sprayed at 60 µL/min displayed a decrease in biofilm formation of 60.6% when compared to the blank control that had no plasma or antibiotic treatment. The higher flow rate of the tobramycin solution at 100 µL/min showed a decrease of up to 73.5% in formation, with the intermediate flow rate of 80 µL/min showing a decrease of 65.5%. The 20 µL/min incremental increase in antibiotic flow rate had a clear impact on bacterial growth, as there was a decrease of 4.9% between the 60 and 80 µL/min flow rates. Similarly, for the 80 and 100 µL/min flow rates, a decrease of 7.9% was observed between the two parameters. Tobramycin wet deposition without plasma proved to be effective against the growth of the biofilms (Figure 7). Statistical analysis showed that all three doses of antibiotic produced a statistically significant reduction in biofilm formation (*p* < 0.05). In addition, the difference between the 60 and 100 µL/min flow rates was also significant (*p* = 0.007).

**Figure 8:**
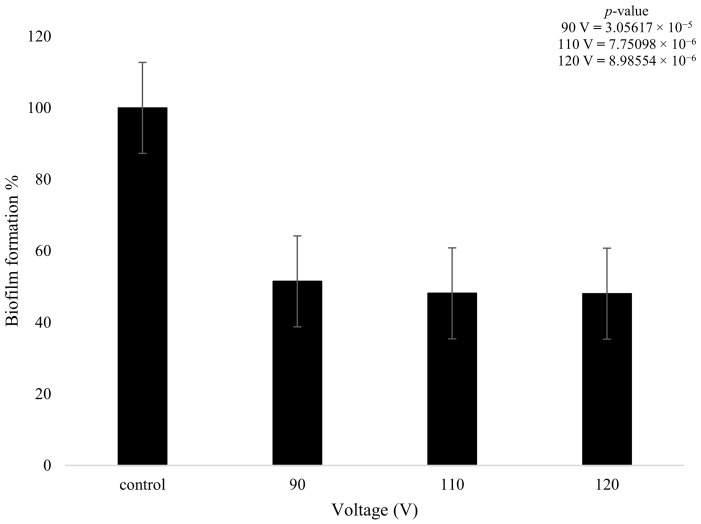
The figure shows *K. pneumoniae* biofilm formation (%) after plasma treatment using 90, 110, and 120 V on the *K. pneumoniae* biofilms (*n* = 32). *p*-value results of the treatments when compared to the control sample that consisted of no treatment included in top right corner.

On the other hand, the biofilms that were treated with plasma only produced a statistically meaningful decrease in growth when compared to the control. Despite the changes in plasma power, all three plasma settings had a similar effect on biofilm formation. The 90 V treatment led to a decrease of 49% in terms of biofilm formation. The higher-power 110 V and 120 V treatments had equivalent results of a decrease of 52%, showing that increasing the voltage did not necessarily result in a measurably higher reduction in biofilm formation. There was a slight decrease (3.28%) in bacterial formation when the voltage was increased from 90 to 110 V and even lower at 0.1% when it was increased from 110 to 120 V (Figure 8); however, these changes are not statistically meaningful. The *t*-test highlighted that the plasma treatment had a major impact on the growth of biofilm formation in comparison to the control, with all values providing a *p*-value below 0.05, as shown in Figure 8.

The plasma pre-treatment consisted of initially treating the biofilms with the plasma discharge, followed by the wet deposition of the tobramycin solution (15 mg/mL) at a flow rate of 60 µL/min. It could be seen that the 90 V and 60 µL/min treatments caused a reduction of 59.63% in biofilm formation, with the 110 V and 60 µL/min parameters leading to a 67.43% decrease in biofilm formation. Lastly, the 120 V and 60 µL/min application showed a similar result to parameter 2, with a reduction of 64.1%.

**Figure 9:**
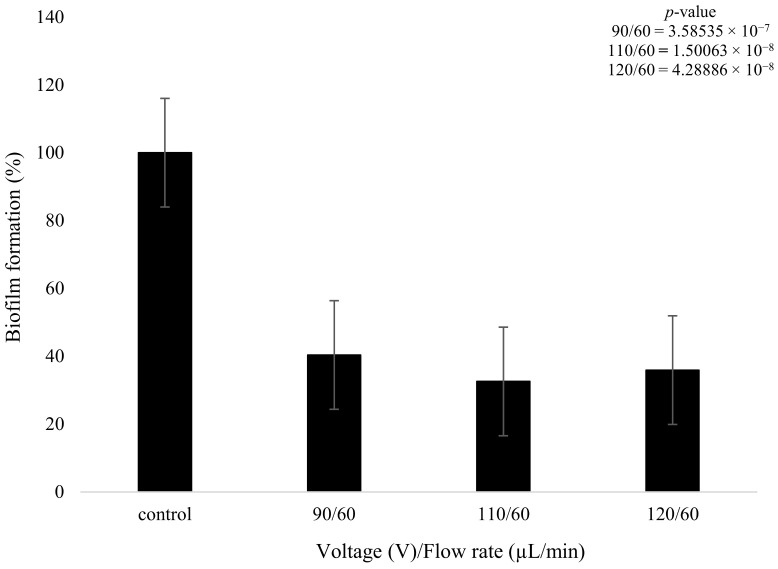
The figure shows *K. pneumoniae* biofilm growth (%) after plasma treatment using 90,110 and 120 V followed by the wet deposition of tobramycin (15 mg/mL) at 60 µL/min (*n* = 32). *p*-value results of the treatments when compared to the control sample that consisted of no treatment included in top right corner.

It was observed that as the plasma pre-treatment increased from 90 to 110 V, there was a further decrease of 8% in biofilm formation (Figure 9). On the contrary, when the plasma pre-treatment was further increased from 110 to 120 V, there was little change in biofilm formation, with an actual increase of 3.9% in formation being observed. The higher plasma power did not necessarily decrease biofilm formation but instead produced a complex effect, with values fluctuating as the power increased.

Further statistical analysis confirmed that all three parameters produced significant effects on the biofilm structures in comparison to the control (*p* < 0.05, Figure 9). However, the changes observed between different voltages were not significant.

### 3.5. Water Contact Angle

#### 3.5.1. Titanium Coupon Surface

It was noted that the plasma and non-plasma deposition on the activated coupon altered the surface energy. The blank uncoated coupon was found to have contact angle measurements of up to 97°, but after plasma activation in the vacuum chamber, the water contact angle (WCA) dropped by 60° (Table 2). The plasma-deposited tobramycin (10 mg/mL) coating showed a 13° reduction in comparison to the activated coupon. However, the wet-deposited coating (10 mg/mL) displayed a higher WCA at 42° than the plasma-deposited coating (10 mg/mL).

Interestingly, the WCA for the plasma and wet-deposited 15 mg/mL coating displayed the lowest surface energy (<5°) compared to the other concentrations.

The 20 mg/mL tobramycin solution also displayed a lower contact angle when compared to the control coupon, with the plasma-deposited sample reaching a low of 14°. The wet-deposited sample (20 mg/mL) displayed a slightly higher WCA than the activated coupon at 36°. Despite the antibiotic coating, the WCA remained low, with the wet-deposited coating presenting a higher WCA than the plasma-deposited samples.

#### 3.5.2. Polystyrene 96-Well Plate Surface

The WCA of the tobramycin non-activated wet deposit (NAWD) plate was taken a day after the coating was applied to display the differences between the activated surface and the activated polystyrene surface. The wet-deposited layered tobramycin coating was conducted on an activated polystyrene plate, whilst the plasma-deposited parameter consisted of plasma depositing the tobramycin onto an activated polystyrene plate (Table 3).

It was observed that the WCA was higher for the NAWD parameter in comparison to the plasma and wet-deposited plates that were activated prior. The wet-deposited coating from layer 1 to 3 ranged from 16 to 20.6°, with the higher contact angle being observed at layer 1. It could be seen that the more layers deposited, the lower the WCA was in relation to the wet and plasma-deposited samples.

In the plasma-deposited coating of tobramycin onto an activated polystyrene plate, it was observed that the WCA of layer 1 started off relatively low before increasing slightly by layer 2. Interestingly, there was a decrease in the WCA of plasma deposit layer 3, which was 10.45°.

Overall, the non-activated wet deposit had the most inconsistent measurements compared to the wet deposit and plasma-deposited plates. The NAWD increased and decreased erratically through the varying layers, which did not present a clear trend, with layer 1 presenting the same value as the layer 3 value. The wet deposit and the plasma deposit had a lower WCA to start off with and did not have any significant increases in the WCA but, instead, decreased by the third layer.

### 3.6. AFM

The surface roughness of the tobramycin-coated coupons, plasma, and non-plasma, at 10, 15, and 20 mg/mL is depicted below in Table 4. The table displays that the tobramycin wet deposition did not majorly impact the surface of the coupon.

The AFM analysis of the wet-deposited coated coupons at 10, 15, and 20 mg/mL overall displayed a lower surface roughness than the uncoated sample. There was a steady increase in surface roughness from the lowest concentration to the highest. Tobramycin wet deposition created a thin layer of coating on the surface, which can be observed through the increase in roughness by 7.7 nm.

The plasma-deposited titanium coupons showed that all the coated samples had lower surface roughness in comparison to the uncoated samples (122.0 nm). The 10 mg/mL plasma-deposited sample had a higher surface roughness than the 15 and 20 mg/mL samples, with the 15 mg/mL sample showing the lowest roughness (42.5 nm). There was a drastic drop in surface roughness from the 10 to the 15 mg/mL samples, but eventually, the roughness plateaued at 44.0 nm.

Overall, the deposition of tobramycin via wet and plasma deposition decreased the surface roughness of the coupon surface. The plasma-deposited tobramycin displayed a lower surface roughness in the case of 15 and 20 mg/mL deposition, whilst the wet deposited sample displayed a gradual increase in surface roughness.

### 3.7. FTIR

The chemical structure of the deposited tobramycin was analysed using FTIR. The control sample was the wet deposition sample, and this was compared against the plasma-deposited sample. The plasma sample used 90 V of power, and both samples involved the static deposition of tobramycin at a flow rate of 60 µL/min. Both the spectra below contained a broad band beyond 3000 cm^−1^, which consisted of an O-H stretch at 3400 cm^−1^. The wet deposition spectra presented a larger band in this region than plasma deposition [23].

This broad region that contained the COOH and NH_3_ groups provided strong peaks at 3166 cm^−1^ (N-H) and 3036 cm^−1^ and aliphatic signals at 2966 cm^−1^. The hydroxyl group present was comprehensive in both spectra in the 1666 cm^−1^ and 1390 cm^−1^ regions. Lastly, the amine group with asymmetrical stretching was observed between the 1027 cm^−1^ and 1034 cm^−1^ regions [23].

**Figure 10:**
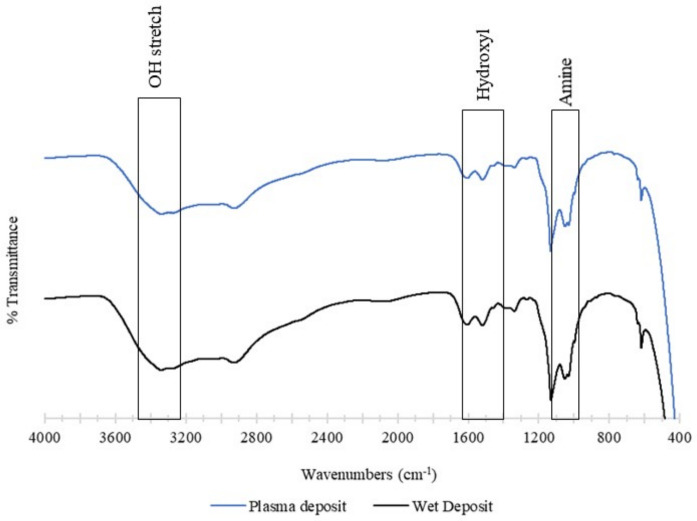
The figure shows FTIR analysis of the tobramycin sulphate deposited at a (top) plasma deposition of 60 µL/min using 90 V and (bottom) wet deposition using a 60 µL/min flow rate and no plasma.

In summary, the functionality of tobramycin was not affected by the plasma deposition. The spectra showed that wet deposition had stronger peaks than plasma deposition, yet this had no effect on the antibiotic’s ability to inhibit *K. pneumoniae* planktonic and biofilm growth. The observation of similar peaks in both spectra demonstrates that the plasma does not cause any alterations to the structure of tobramycin (Figure 10).

## 4. Discussion

Although there are many preventative measures in place to reduce the risk of infection in hospital settings, implants and surgical sites still become infected. These infections are caused by prevalent resistant strains of bacteria, also known as ESKAPE pathogens [14]. These pathogens are a major issue due to their resistance, which include the formation of a biofilm layer around an aggregate of colonies, making them harder to eradicate [11].

In this study, a plasma deposition system was used on titanium coupons and polystyrene 96-well plate surfaces and directly onto biofilm formations. This was carried out to demonstrate that the BioDep deposition system can be used to deposit an antibiotic alongside the plasma without a loss in antibiotic functionality. Tobramycin was used in this study due to its known effectiveness against *K. pneumoniae.*

The minimum amount needed to reduce *K. pneumoniae* growth was observed and found to be 120 µg/mL, which is 0.12 mg/mL. This value was taken into consideration in the coating process. In order to observe differences in growth due to the plasma, the antibiotic deposited needed to be below 0.12 mg/mL for all of the parameters investigated. Depositing amounts in excess of this would have rendered it impossible to observe the changes in bacterial growth, as total eradication would not display differences due to the plasma parameters.

In addition, the planktonic colony growth of *K. pneumoniae* was also observed over a span of 6 h. These hours are particularly critical, and if colonisation can be inhibited during this critical period, then infection is much less common on implanted devices [24,25]. The results were promising, as it could be seen that there was a decrease in the colony formation of the antibiotic-coated polystyrene plate, whilst the blank sample had a high CFU/mL. The 6 h period was essential to monitor as preventing the planktonic colonies from forming in the exponential phase can decrease the chances of a biofilm structure growing [24].

The effect of surface energy was also investigated in this study as this can impact bacterial adhesion [26]. Simple plasma activation was used to create an activated or hydrophilic surface, while the untreated substrate acted as a hydrophobic surface [27]. It could be seen that the activated surface of the wet-deposited and plasma-deposited tobramycin had a slightly lower CFU/mL value in comparison to the non-activated wet deposit plate. Therefore, the activated surface was found to contribute to the efficacy of the process. It was theorized that the activation process would render the surface hydrophilic, and this may have decreased the affinity and attachment of hydrophobic bacteria [26,28]. A study carried out by Trentin et al. in 2014 displayed that a more hydrophilic surface was more effective in inhibiting the adhesion of *K. pneumoniae* bacterial colonies, up to 83% [29].

The surface analysis of the titanium coupons (Table 2 and Table 4) also supported this concept. Water contact angle testing showed increased hydrophilicity across all the samples in comparison to the blank coupon. It was noted that despite varying the antibiotic concentration, there was little to no difference in the wettability of the coated samples. This may suggest that complete coverage of the surface was obtained even with low doses of antibiotics and that further doses only increased the thickness of the coating and not the coverage. It could also be seen that the combination of the plasma and antibiotic deposition resulted in a lower WCA, with the wet-deposited samples showing similarities to the activated coupon due to the liquid layer.

The observation of antibiotic functionality after being deposited onto the Ti coupon surface showed that tobramycin was still chemically intact and effective after deposition. Increasing the concentration of antibiotic on the surface could be achieved either by increasing the concentration of tobramycin in the initial liquid or by building up multiple layers of deposited antibiotic. Both methods of increasing the antibiotic load produced related results, which shows that the antibiotic dose can be controlled through layering whilst retaining good efficacy against the planktonic cells. The prevention of planktonic cell attachment is important as they instigate the initial adhesion onto the surface that leads to the micro colony [30]. These initial stages after implantation are extremely important as the host immune system is weakened [31] and can allow a microbe to win the race to the surface [24]. This layer of antibiotics on the biomaterial can give the host the opportunity to recover after surgery by delaying microbial contamination and allowing the host tissues to integrate with an implant, as has been shown through the use of antibiotic-loaded cements in orthopaedics [32].

Despite best efforts to prevent infections during surgery, implants still become infected and remain difficult to treat [11]. In the case of the *K. pneumoniae* biofilms exposed to tobramycin, it was observed that there was a significant decrease in biofilm formation, as was expected. Increasing the concentration of antibiotics further decreased the viability of the biofilm. Similarly, the plasma treatment alone also decreased the viability of the biofilm, though not as effectively as the pharmaceutical intervention. Increasing the voltage of the plasma further did not produce any meaningful enhancement in terms of biofilm eradication in this study.

Combining the two interventions led to complex interactions. The combination of low antibiotic flow and low voltage produced a biofilm reduction that was equivalent to just the antibiotic effect on its own. At that level, it appears that the additional plasma has no impact. When the low flow of antibiotic was combined with a higher voltage plasma, an additional reduction in biofilm viability was observed. Interestingly, increasing the plasma power further did not produce the expected result. Instead of further decreasing biofilm viability, it led to a less effective kill mechanism and actually reduced the efficacy of the antibiotic. This may suggest that at very high plasma powers, the plasma can begin to degrade the antibiotic, thereby counteracting the benefits of combining the two treatments [33]. The optimal plasma and antibiotic parameters were therefore determined to be 110 V and a 60 µL/min flow rate of tobramycin.

This study effectively displayed that the plasma deposition of tobramycin was effective after the deposition process and worked well against *K. pneumoniae* in its planktonic and biofilm form. The antibiotic was also still effective after deposition on the Ti coupon, but further investigations would have to be carried out to observe the retention efficacy of tobramycin as well as the synergistic effects of CAP with other kinds of antibiotics.

The prospect of this study would be to utilize the plasma deposition system to treat implant surfaces prior to surgery to achieve a sterilized and antibiotic-coated surface. This can be useful in the cases of patients who cannot systematically receive antibiotics to the surgical site due to poor perfusion or other issues. The antibiotic-coated implant can prevent the need for systemically administering antibiotics due to the fact that the site is locally treated. This study also shows a significant decrease in biofilm formation as the plasma pre-treatment is applied before the antibiotics. The synergistic effects of these two parameters can aid in the treatment of infected surgical sites and open wounds.

## 5. Conclusions

In conclusion, the plasma deposition of tobramycin showed significant efficacy in reducing the percentage of biofilm growth as well planktonic bacteria growth of *K. pneumoniae*. The deposited antibiotic on the titanium coupon surface remained active and created zones of inhibition on the bacterial lawn, with there being a minor difference between the 5 mg/mL incremental increases in antibiotic concentration. The direct treatment of exposed *K. pneumoniae* biofilms using deposited tobramycin was effective in decreasing biofilm formation, and the effectiveness could be increased when plasma was used to treat the biofilms prior to the antibiotic treatment. This study presents an alternative to using higher concentrations of antibiotics by substituting the higher dose with a suitable plasma power. Decreasing antibiotic load can have desirable effects in the future as the risk of systemic exposure to antibiotics can be reduced by simply treating the infected area with the plasma before antibiotic administration, which can improve the outcome and risks associated with IAIs.

## Figures and Tables

**Table 1:** The table shows *T*-test analysis of two-tailed variance between the blank sample with no treatment and the treated parameters, as listed below.

*Parameters*	*0 h*	*2 h*	*4 h*	*6 h*
*Non-activated wet deposit (NAWD)*	0.50	1.28 × 10^−13^	3.99 × 10^−9^	9.86 × 10^−10^
*Wet deposit*	0.53	2.34 × 10^−11^	8.51 × 10^−8^	3.59 × 10^−7^
*Plasma deposit*	0.39	1.52 × 10^−13^	8.58 × 10^−9^	4.58 × 10^−8^

**Table 2:** The table shows the water contact angle (WCA) measurements of the plasma and non-plasma-deposited tobramycin (10, 15 and 20 mg/mL) on activated titanium coupon surface. Measurements recorded on the Kruss TVA100.

Sample	WCA (°)
Control (blank coupon)	97
Activated coupon	31
Plasma-deposited (10 mg/mL)	18
Wet-deposited (10 mg/mL)	42
Plasma-deposited (15 mg/mL)	<5
Wet deposited (15 mg/mL)	<5
Plasma-deposited (20 mg/mL)	14
Wet-deposited (20 mg/mL)	36

**Table 3:** The table shows the water contact angle (WCA) values of the polystyrene plates coated with tobramycin (10 mg/mL) under different conditions.

	Non-Activated Wet Deposit (NAWD) (°)	Wet Deposit (°)	Plasma Deposit (°)
**Layer 1**	34.7°	20.6°	16.4°
**Layer 2**	38.1°	19.7°	18.8°
**Layer 3**	34.3°	16.0°	10.5°

**Table 4:** The table shows AFM analysis of the wet and plasma-deposited tobramycin-coated coupon at 10, 15, and 20 mg/mL, displaying values for the average surface roughness (nm).

Tobramycin (mg/mL)	10	15	20	Uncoated
**Wet-deposited**	55.0 nm	61.9 nm	62.7 nm	122.0 nm
**Plasma-deposited**	88.5 nm	42.5 nm	44.0 nm	122.0 nm

## Data Availability

The original contributions presented in the study are included in the article, further inquiries can be directed to the corresponding author.
